# A survey: Precepts and practices in drug use indicators at Government Healthcare Facilities: A Hospital-based prospective analysis

**DOI:** 10.4103/0975-7406.76502

**Published:** 2011

**Authors:** Hettihewa L Menik, Amrasinghe I Isuru, Subasinghe Sewwandi

**Affiliations:** Department of Pharmacology, Faculty of Medicine, University of Ruhuna, Sri Lanka, Molecular Science and Biomedical Unit, Faculty of Medicine, Sri Lanka; 1 Allied Health Science degree programme, Faculty of Medicine University of Ruhuna, Sri Lanka

**Keywords:** Drug use pattern, essential drug list, polypharmacy, prescriber errors, rational prescription

## Abstract

**Background:**

We planned to identify the difficulties in practicing the rational use of medicine in health facilities, using drug-use indicators.

**Materials and Methods:**

We studied the average consultation time (ACT), average number of drugs per encounter (ANDE), percentage of drugs by generic name (PDPG), percentage of encounters with antibiotics (PAP), percentage of encounters with injection (PIP), percentage of drugs prescribed from the essential drugs list (PEDL), using pretested questionnaires in different hospital types.

**Results:**

There was a higher value of ACT in Teachin hospital (TH,2.31 min) and general hospital (GH,2.17 min) compared to district hospital (DH,0.83 min). ANDE was high in all three categories (3.24, 2.88, and 3.26 in TH, GH, and DH, respectively). There was a significant difference in ANDE in all three categories (*P*≤0.05). There was no significant difference in the PDPG among all categories of Hospitals. PAP was highest in DH (80%) and lowest in GH (46%). PIP was highest in DH (6%), 4% in GH, and lowest in TH (3%) in the Galle district. PEDL in TH, GH, and DH were 97, 100, and 99%, respectively. Prescribers use a short consultation time and practice polypharmacy, and the use of generic and essential drug lists is significantly high. Antibiotic usage is high, but usage of injections is low. We further noted prescriptions with absence of the diagnosis, sex, and prescriber’s identity.

**Conclusion:**

: We conclude that some areas like polypharmacy, high usage of antibiotics, and poor prescription writing practices are high and they can be addressed by in-service awareness programs for noted prescriber errors.

Good prescribing practice is an essential part of rational drug use.[1,2] A prescription audit, therefore, is a useful method to assess the doctors’ contribution to rational use of drugs in a country. Different aspects of prescribing patterns in many institutions, in different countries like India,[3–7] Pakistan,[8,9] Nepal,[10] and Sri Lanka[11,12] have been studied.

We found that there was limited data available about the inappropriate prescription practices such as polypharmacy and over-usage of antibiotics and injections in the government hospitals in Sri Lanka. In the absence of more data in this field, we decided to study the degree of a healthcare worker’s adherence to principles of rational use of medicine in the public sector initially in Galle, Sri Lanka. We planned to measure the prescribing indicators including polypharmacy, prescription of generics and essential drugs, injections / antibiotic usage, patient care indicators, and the average consultation time, to assess the adherence to the rational drug policy. We decided to use the specific parameters like average consultation time (ACT), average number of drugs per encounter (ANDE), percentage of drugs prescribed by generic name (PDPG), percentage of encounters with an antibiotic prescribed (PAP), percentage of encounters with an injection prescribed (PIP), and percentage of drugs prescribed from the essential drugs list or formulary (PEDL for our analysis because these were the standard parameters recommended by the World Health Organization (WHO) for analysis of drug-use patterns (13).

It was determined to address the recognized issues at the local and national level by giving our recommendations for improvement.

## Materials and Methods

It was a study carried out at the TH, Karapitya, GH in Balapitiya and DH in Akuressa for six months. All patients attending the Outpatient Departments in the morning clinics in theseh were considered and included in our study. The ethics and review committee of the institution approved the study. Five hundred and ninety encounters were collected from patients who attended the OPD, by the trained medical students and medical officers. Data collectors were pre-trained by a principle investigator, in an effort to ensure uniformity in data collection. This prescriber care assessment was done by doing exist-patient interview using a pretested structured observations questionnaire. The following measuring tools were used to assess the degree of prescriber care and the errors in our health facilities. ACT, ANDE, PDPG, PAP, PIP, and PEDL were calculated for three different hospitals according to the WHO criteria.[13]

## Results

Patient diagnosis and prescriber identity were absent in all the prescriptions, although the signature was present in almost all. Age was not mentioned only in 0.61% patients, but sex was not mentioned in any of the 327 prescriptions studied. Duration of treatment and frequency of drug administration were also not mentioned in 0.61% of the prescriptions [Table 1].

**Table 1:** Common problems noted in the prescriptions analyzed in TH, GH and DH in Galle district

Problems encountered	Percentage of prescriptions
Diagnosis is absent	100
Doctors identity is absent	100
Age is not mentioned	0.61
Sex is not mentioned	100
Duration of the treatment	0.61
Frequency of drug administration	0.61

### Significant differences in average consultation time, in different hospital set-ups, in the Galle district

**Figure 1:**
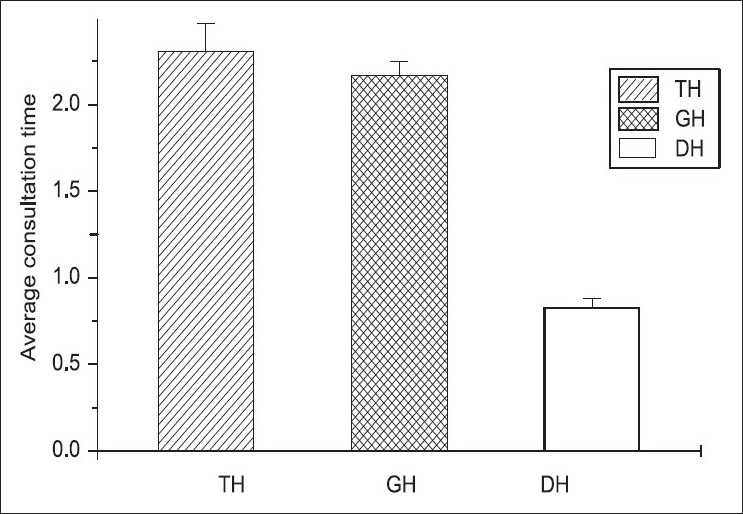
Average consultation time (ACT) in different hospitals using ANOVA. Shows the changes in ACT in different hospitals and we observed the ACT value to be 2.31±0.16 in TH, 2.17±0.08 in GH, and 0.83±0.05 in DH, respectively, M±SEM. The three means are significantly different (*P*≤0.05)

We analyzed 263 prescribers in government hospitals using the pretested structured observations questionnaire used to assess patient care by WHO. The average consultation time (ACT) of three different hospitals is given in Figure 1.

### Average number of drugs per encounter

**Figure 2:**
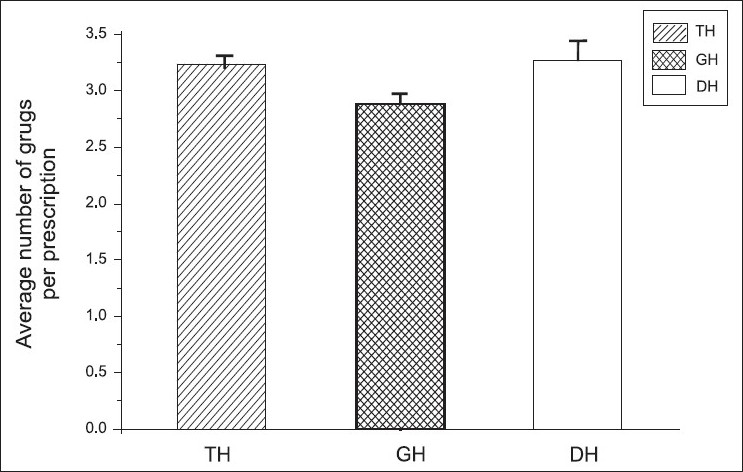
Average number of drugs per encounter (ANDE) in different hospitals using ANOVA. Shows that the changes in ANDE in different hospitals and the ANDE value are 3.24±0.08 in TH, 2.88±0.10 in GH, and 3.26±0.17 in DH, respectively, M±SEM. The three means are significantly different (*P*≤0.05)

Mean ± SEM of average number of drugs per encounter (ANDE) is given in Figure 2. According to the WHO, 2008, recommended figures (1.6 – 1.8), our ANDE was very high. We further noted that ANDE was high in our DH and TH and relatively low in GH.

### Percentage of drugs prescribed by generic name

**Figure 3:**
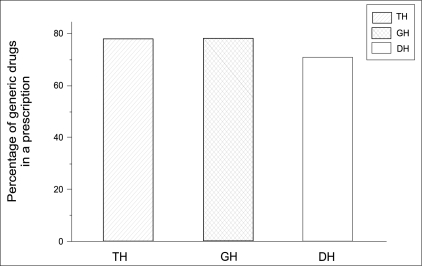
Percentage of drugs prescribed by generic name (PDPG) in different hospitals using ANOVA. Figure 3 shows the changes in PDPG in different hospitals and we observed the PDPG value as 78% in TH, 78% in GH, and 71% in DH, respectively

Table 2 shows average number of prescribed generics per prescription in different hospitals. Our results showed that percentage of drugs prescribed by generic name (PDPG) was lower than the WHO recommended values and use of generic names in the government prescriptions was significantly high. It was also noted that PDPG was high in TH and GH [Figure 3]. It was low in DH where only the basic facilities were available. We also found that there was no significant difference in the average number of drugs prescribed by generic name among the three groups of hospitals (* P*≤0.05).

**Table 2:** Average number of generics per prescription

Hospital	Mean ± SE
TH (Teaching Hospital)	2.54 ± 0.08
GH (General Hospital)	2.26 ± 0.10
DH (District Hospital)	2.31 ± 0.14

### Percentage of encounters with an antibiotic prescribed

**Figure 4:**
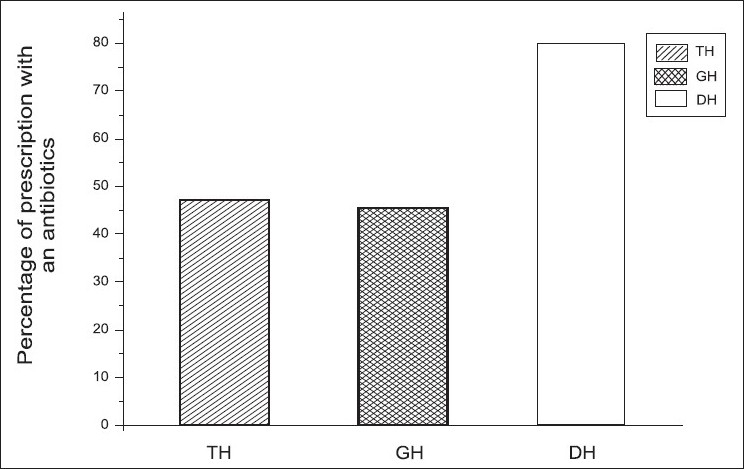
Percentage of encounters with an antibiotic prescribed (PAP) in different hospitals using ANOVA. It shows the changes in PAP in different hospitals and we observed the PAP values as 47% in TH, 46%in GH and 80% in DH, respectively

Different values of Percentage of encounters with an antibiotic prescribed (PAP) in TH, GH, and DH are given in Figure 4. We found that the percentage of antibiotics was very much higher than the WHO recommended values (20 − 26.8%). It was observed that PAP was comparatively low (47%) in TH and high (80%) in DH.

In addition to the percentages of antibiotic usage, three means of the average number of antibiotics per encounter in TH (47 + 0.04), GH (46 + 0.07), and DH (80 + 0.09) were also compared. We found that the DH mean was significantly different from GH and TH (*P*≤0.05, Table 3).

**Table 3:** Average number of antibiotics per prescription

Hospital	Mean ± SE
TH (Teaching Hospital)	0.55 ± 0.05
GH (General Hospital)	0.58 ± 0.07
DH (District Hospital)	1.11 ± 0.12

### Percentage of encounters with an injection prescribed (PIP)

We found that percentage of injections was lower in our study than the WHO recommended values (13.4 −24.1%). Use of injections in government prescriptions was significantly low.

**Figure 5:**
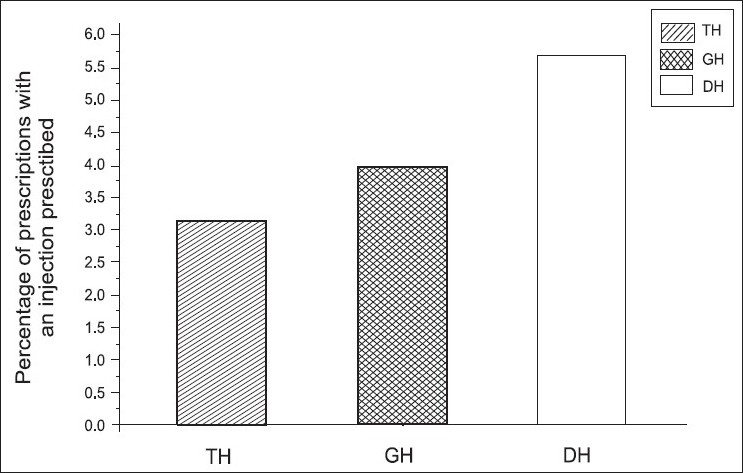
Percentage of encounters with an injection prescribed (PIP) in different hospitals using ANOVA. Figure 5 shows the changes in PIP in different hospitals and we observed the PIP values to be 3% in TH, 4% in GH, and 6% in DH, respectively

In addition to percentages, we further analyzed the average number of injections per encounter in these hospitals [Figure 5], and the study showed that there was no significant difference in the average number of injections per prescription among the three groups of hospitals (*P*≤0.05, Table 4).

**Table 4:** Average number of injections per a prescription

Hospital	Mean SE
TH (Teaching Hospital)	0.03 ± 0.01
GH (General Hospital)	0.04 ± 0.02
DH (District Hospital)	0.06 ± 0.04

### Percentage of drugs prescribed from essential drugs list or formulary

**Figure 6:**
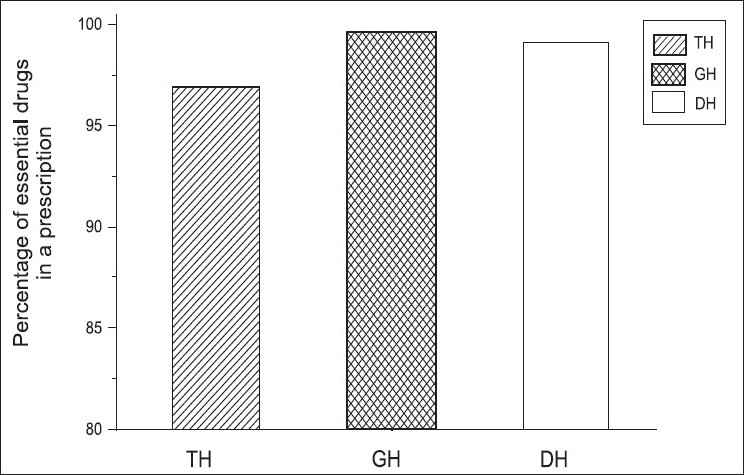
Percentage of drugs prescribed from essential drugs list or formulary (PEDL) in different hospitals using ANOVA. Figure 6 shows the changes in PEDL in different hospitals and we observed the PEDL value to be 97% in TH, 100% in GH, and 99% in DH, respectively

Table 5 shows average number of drugs prescribed from essential drug list three different hospitals. Figure 6 shows the percentages of Percentage of drugs prescribed from essential drugs list or formulary (PEDL) in TH, GH, and DH, and we found that the percentages of drugs prescribed from the essential drugs list or formulary were compatible with the WHO values (100%). Our PDEL in the government prescriptions was significantly high.

**Table 5:** Average number of drugs written from EDL or formulary per a prescription

Hospital	Mean ± SE
TH (Teaching Hospital)	3.14 ± 0.08
GH (General Hospital)	2.87 ± 0.10
DH (District Hospital)	3.22 ± 0.17

In addition to the percentage of essential drugs prescribed from the essential drug list or formulary, the average number of drugs used by the EDL or Formulary per encounter in these hospitals was also studied. We also found that there was no significant difference in the average number of drugs prescribed from the EDL or Formulary among the three groups of hospitals (*P*≤ 0.05).

## Discussion

The drug prescribing pattern needs to be evaluated from time to time.[14] Our health system mainly consists of government hospitals in all places, and treatment and other health services are given free to the population. There is a minor population who seeks private sector service, mainly in the city limits. Therefore the healthcare system and services provided by the government system bear a majority of the weight in the population when compared to other countries, where both private and public sectors have an equal burden.

We found that the consultation time of a prescriber in government hospitals is very much shorter when compared with that in private practice.[12,15] ACT in Nepal was higher than in our study (6.03 ± 3.34 minutes). The short ACT can be explained by inadequate patient doctor ratio in our public sector free health service.

We also found various prescriber problems noted on the 327 prescriptions. These prescriber problems were identified and listed in Table 1. Although the diagnosis was an essential component in a prescription, it was not written in 100% of the prescriptions, in all the screened hospitals. This was one of the common errors seen in the government hospitals in Sri Lanka.. This finding again could be justified with the large number of patients alloted to a single medical officer. These issues should be critically addressed by the government to reduce all the complications, like adverse reactions and high health cost, and the morbidity and mortality rate in the country. Our results showed that both frequency of drug administration and duration of treatment were indicated satisfactorily, 100, 100, and 94.29% of the prescriptions in TH, GH, and DH, respectively. We also found that the duration of the drug treatment was also mentioned in 100, 100, and 94.29% of the prescriptions in TH, GH, and DH, respectively.

We analyzed the degree of adherence to the use of EDL and STG in government hospitals by using the WHO standard in the recommended evaluation techniques. Our study showed that the use of essential drugs for prescriptions were parallel with the WHO standards. in the studied hospital categories. This practice could be due to the government health policy of EDL, and it was helpful to reduce the national health budget of the country and hence improve the cost-effective strategies.

This study further showed evidence for a great need to improve prescription writing, as evidenced by our data, where in some of the prescriptions, duration of treatment, and frequency of drug administration were not given. This problem coupled with polypharmacy could result in less safe, more expensive. and irrational prescribing.[16]

We have noticed that doctor identity is absent in all the prescriptions and it will be a serious dilemma in incidences where the prescriber is needed for an emergency. We feel that this is because of complete negligence. Despite the work load, the importance of this should be stressed, as both the patient’s and doctor’s identities are vital on a prescription in Sri Lanka.

We further noted that prescription of injections at the OPDs set up in the government hospitals was very low, but parallel to the WHO standard[17] and could be explained by the high turnover of patients in the OPD setup. It was a similar case with the establishment of emergency treatment units at the OPD. In contrast to our results, in the research conducted in the Manipal teaching hospital, the percentage of injections was 5.21% of the encounters.[3] According to them, the percentage is more than that in the Pakistani study.[3] In contrast to that, the prescription for antibiotic use in our hospitals is very high, in all hospitals types, when compared to the WHO values, and this should be addressed as early as possible, to prevent adverse reactions due to antibiotic use.

The average number of drugs per prescription is an important index to review, to plan an educational intervention in prescribing practices. We found that the number of drugs per encounter in all hospital categories was higher than the WHO recommended values (1.6 – 1.8). Similar results were found in Cambodia, Ethiopia, Morocco, Tanzania, Zimbabwe, and Nepal.[14]

Other hospital-based studies[10] in India also reported figures of three to five drugs per prescription, which was quite similar to ours.[11] Various reasons can account for this deviation from the recommended WHO values. It can be due to unrealistic expectations, quick relief from patients, common practice of irrational drug combinations, unnecessary use of vitamins, and aggressive medicine promotions. This concept, which is called as polypharmacy, can lead to increased risk of drug interactions,[16] increased hospital cost, and errors in prescribing.[18–21]

Our study had some limitations. We did not plan to interview patients for their knowledge of taking the correct doses. This is important, because the absence of the knowledge on correct drug administration will lead to poor achievements of rational practice. This study needs to be followed up by prescriber education on rational drug use, by means of short-term training sessions, including a briefing on proper prescription writing. We further plan to do a reaudit, to measure the impact of intervention.

We are happy to see that a maximum number of drugs from the EDL has been used in all three hospitals in the Galle district and we understand that it is a common practice in most of the government hospitals in Sri Lanka. This is probably because procurement has to be done according to successful implementing plans and procurement policies. One research shows only 7.61% of the prescribed generic drugs in a private hospital in their study. We also feel that usage of EDL might be low in the private sector in Sri Lanka, however, this needs to be investigated.

To achieve a good goal of rational use it is not sufficient to choose the right medicine — health workers must be employed in the most appropriate proportion. We have noticed that most of the errors found in our study are related to very low doctor / patient ratio even in the tertiary care hospital. Therefore, to minimize the prescribing errors, we recommend the administrative authority in the government to consider increasing the doctor/ patient ratio. To reduce the complications of polypharmacy and improve the rational practice we recommend that the prescribers keep the number of medicines to the lowest and prescribe only those that are necessary. Please include this message.

### Conflict of interest

We hereby disclose that the study which we carried out under this drug-use pattern in the Galle District does not have any type of financial or personal relationship with other people or organizations that could inappropriately influence (bias) our study.

We received a small grant from the university, under the Improving Relevance and Quality of Undergraduate Education (IRQUE) project, to buy stationery and for day-to day expenditure.
